# A new custom-made bivalve brace for pectus carinatum in children and adolescents: preliminary promising experience of 140 patients from a tertiary center

**DOI:** 10.3389/fped.2024.1321633

**Published:** 2024-04-02

**Authors:** Simone Frediani, Angelo Zarfati, Valerio Pardi, Ivan Aloi, Arianna Bertocchini, Antonella Accinni, Federico Beati, Massimiliano Pasanisi, Alessandro Inserra

**Affiliations:** ^1^ General and Thoracic Pediatric Surgery Unit, Bambino Gesù Children’s Hospital, IRCCS, Rome, Italy; ^2^ University of Rome “Tor Vergata”, Rome, Italy

**Keywords:** pectus carinatum, conservative treatment, children, thoracic wall malformation, custom-made bivalve brace

## Abstract

**Introduction:**

International research suggests that poor patient compliance is the main cause of tutor failures in the context of potential novel orthopedic bivalve braces for conservative treatment of pectus carinatum. Our entire experimental study is based on the hypothesis that a rigid bivalve brace that patients can accept could solve the main problem associated with the conservative approach—poor compliance. The hypothesis was to reduce the thickness and weight of the classic bivalve brace to ensure concealment and make it sustainable enough to be worn several hours a day without compromising its therapeutic efficacy.

**Materials and method:**

The research was conducted from January 2020 to December 2022 to ensure follow-up of all participants for at least 6 months. In 36 months, 140 patients with pectus carinatum were assessed and conservatively treated with the studied guardian to analyze the therapeutic efficacy of the bivalve brace and patient compliance. From the initial visit, the parents and patient were informed that this is a 2-year therapeutic course during which the bivalve brace should be worn at least 23 h a day (with 1 h of abstinence per day for routine personal hygiene practices). Compliance is the key to therapy success, and the duration of treatment depends on patient adherence.

**Results:**

The exceptional effectiveness of the experimental brace was confirmed by both the questionnaire from the patients (with an average satisfaction rate of 8.9/10) and an assessment of the therapy's results by a properly selected medical committee (with a VAS scale satisfaction of 7.2/10 for symmetric forms and 7.1/10 for asymmetric ones).

**Conclusion:**

In conclusion, the analyzed data confirmed the research hypotheses. First, none of the 140 patients had cardiovascular diseases directly related to their condition, confirming that pectus carinatum is a pathology of a purely aesthetic nature. Second, a cheap, lightweight, and easily obscured brace significantly improved patient compliance. Along with this, the social relevance of the aesthetic aspect today may be an important factor in motivating the study cohort to adhere to therapy. In the past, esthetics and appearance were less relevant at the social level, which may have contributed to the high abandonment and reduced compliance rates of the many studies in the literature.

## Introduction

Based on the international literature on possible new orthopedic bivalve braces for conservative treatment of pectus carinatum, it was found that the main problem related to the failures of the various tutors proposed in the literature was related to poor patient compliance. Initially, the problem was the difficulty of hiding these devices under clothing, as they were very rudimentary, bulky, and stiff and had unremovable plaster casts. In addition, a limitation of these devices was that they were very heavy and unmanageable, resulting in excessive inconvenience. A major attempt to solve the problem of compliance was the dynamic compressor initially proposed by Haje and Raymundo in 1979 (1) and culminated with the FMF-DCS of Martinez-Ferro in 2008, through which it solved the problems related to the thickness, weight, and excessive pressure exerted by the previous tutors, but at the expense inconsistent optimal results (2).

This study is therefore conducted to address the problem of compliance without falling short of the primary objective, namely, the eradication of the malformative defect. From this perspective, the idea emerged to develop an orthopedic bivalve brace made of a material that combines lightness and durability, with a thickness of a few millimeters to enable easy concealment. This innovative material is called Vivak and allowed us to create an orthopedic bivalve brace model that meets our needs and is easily customizable based on the specific defects of our patients.

In the end, the basic hypothesis on which our entire experimental study is based is that by ensuring an effective treatment through a bivalve brace that, although rigid, possesses properties such that it can be accepted by patients, the core problem of the conservative approach, namely, poor compliance, could be solved. In particular, it was hypothesized to reduce not so much the overall size of the classic bivalve brace, but rather its thickness and weight, to ensure the possibility of concealment and to make it a sustainable weight to be worn several hours a day without difficulty, but without decreasing its therapeutic effectiveness.

## Materials and methods

**Figure 1:**
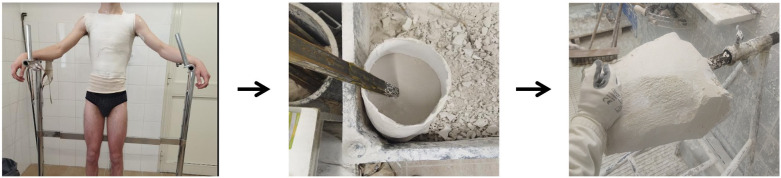
A plaster cast of the chest of the patient (or negative model) is taken and used as a mold to create a positive model, also in plaster, which faithfully reproduces the chest of the patient

**Figure 2:**
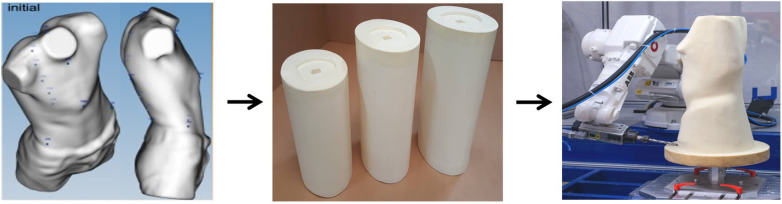
The images obtained with the three-dimensional (3D) scanner are processed by specific software (a negative model) and used to provide information to a robotic cutter, which will obtain a positive polyurethane model.

The study began in January 2020 and ended in December 2022 to ensure that all patients in the study were followed up for a minimum of 6 months. During this 36-month period, 140 patients diagnosed with pectus carinatum were examined and treated conservatively using the examined guardian, assessing both the therapeutic effectiveness of the bivalve brace and its close correlation with the compliance of the patients themselves. From the initial visit, the parents and the patient were informed that this is a 2-year therapeutic course, during which the bivalve brace should be worn at least 23 h a day (considering 1 h of abstinence per day for routine personal hygiene practices). This aimed to clarify how compliance is the real key to therapy success and to emphasize that the overall duration of treatment is also strictly dependent on the degree of adherence of patients to therapy. This new experimental approach to the conservative therapy of pectus carinatum is based on the use of a custom-made bivalve brace, i.e., customized according to the type of defect of the patient, which will selectively compress the protrusion area of the defect. The bivalve brace behaves like a sternal pressure, which will print a compressive force in the rear-front direction on the chest to resolve the deformity both as carenatura and as sternum rotation in cases of asymmetric deformities. Although each bivalve brace is specifically calibrated for the type of defect to be corrected, its underlying structure remains the same, changing exclusively the kind of push it will print on the sternal–costal complex of the different patients. The term “bivalve brace” already identifies the type of brace used, i.e., based on two valves, front and rear, which are connected to each other either laterally through two straps on each side or above through a pair of velcro straps, built by a prosthetist. The valves are made using a plastic material similar to plexiglass, formed by a copolyester with high thermoplasticity, Vivak. It is sold in the form of rigid plates with high transparency, which, precisely thanks to this thermoplastic property, can be processed to take the desired shape, i.e., a shape that recalculates the trunk of the patient in the most faithful way possible. In addition to transparency and thermoplasticity, Vivak has numerous other properties that justify its enormous advantages over other similar materials, including remarkable lightness, exceptional shock and break resistance even at low temperatures, and resistance to atmospheric agents and UV rays. The primary prerequisite for making a tailored orthopedic bivalve brace is to obtain a faithful reproduction of the chests of patients to customize therapy for each of them. Therefore, the personalization of the bivalve brace can be obtained in two ways: obtaining a gips-calm of the trunk of the patient (Figure 1), which will then be processed and used as a mold for the realization of sternal pressure, and using the 3D scanner (Figure 2), through which a computerized image that reproduces the anatomy of the patient in millimeters will be obtained and will be exploited to provide the information to a robotic freezing system capable of making a polyurethane mold of the chest of the patient, which in turn will be used as a basis for the processing of the bivalve brace.

Both modes are extremely reliable, and each has many advantages and disadvantages. Gips is the most used method, as it is a simpler material to work with than polyurethane and cheaper, and orthopedic technicians have more practicality to work with it than any other material. On the contrary, it is less accurate in reproducing the chest mold, requires greater collaboration from the patient, and takes a longer time to realize a negative model. The 3D scanner, on the other hand, is a more elaborate and cutting-edge system, which is extremely precise and allows a quick study of the chest shape of patients. However, it is a more expensive system (requiring, among other things, the use of a robotic mill), and the polyurethane mold is not easy to work, which is why, in the end, the production times of the bivalve brace become overall greater than the times spent with gips. In conclusion, to date, it is still preferable to use the “classic” mode in gips, either because it is preferred by the orthopedic technicians who will then make the bivalve brace or because it is cheaper and the results obtained are still excellent.

Focusing now on the realization of the bivalve brace using the classic technique, it requires a series of procedures, synthesizable in the following stages:

• objective evaluation of the patient to identify the type of carination and the specific correction margin of that patient;
• implementation of the negative model;
• implementation of the positive model based on the negative model;
• implementation of the positive model;
• realization of the bivalve brace, using the positive model as a mold; and
• installation of the compression pad and final test on the patient.

**Figure 3:**
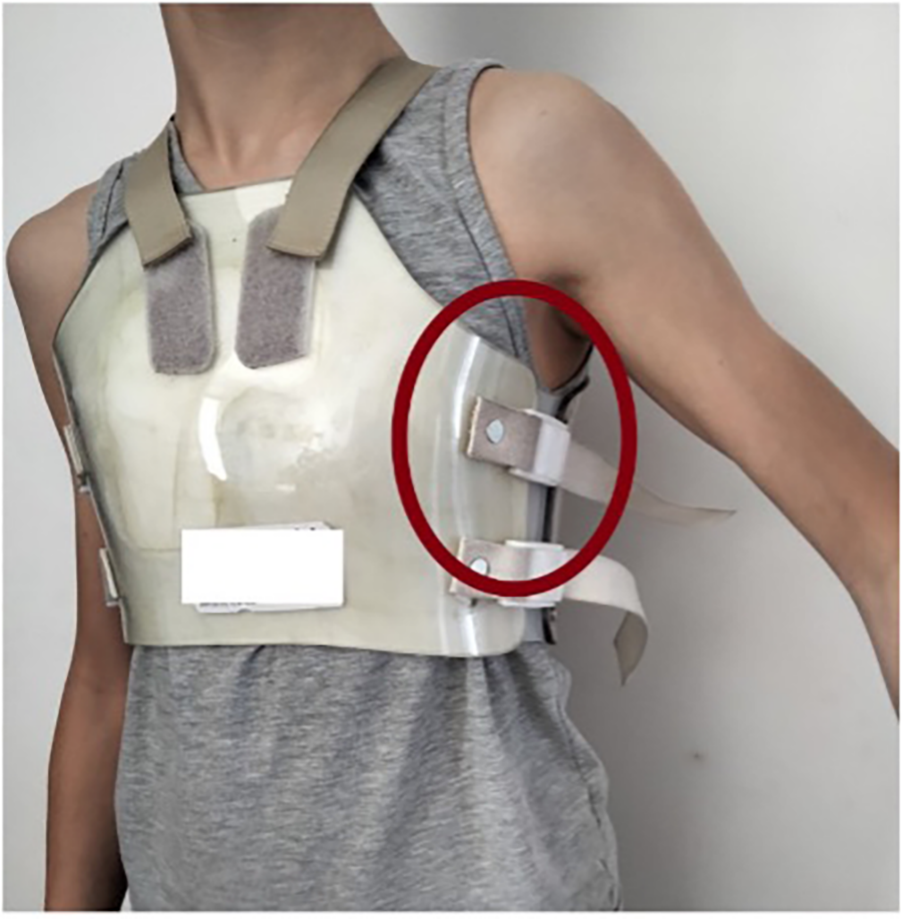
Bivalve brace.

Therefore, for patients with pectus carinatum who are recommended sternal pressure by their chest surgeons, the first step is to visit an orthopedic healthcare provider specializing in this procedure. During a visit, the technician inspects the patient and analyzes his chest deformity. Firstly, it is necessary to identify the type of carenatura of the patient and its severity and then proceed with manual compression of the same to determine the margin of correction, that is, how much push we can print on the patient through the bivalve brace being created. This simple objective assessment is in fact of great importance in the process of realizing the bivalve brace; as we will see later, the greater the correction margin, the more compression we can print on the bivalve brace. Once the first phase is completed, the patient is instructed to wear a net shirt (or shaved shirt) onto which the chest deformity is outlined. Subsequently, the patient is asked to stand as firmly as possible while holding onto a support pad (Figure 3).

Only at this point are the plaster strips immersed in water and then applied to the trunk of the patient, ensuring they are well attached. In fact, after finishing the various plaster bandages, you will get what we call a “negative model,” that is, a faithful reproduction of the chest of the patient. The more adhesive the bandage, the more accurate we will be in achieving an accurate calculation of his chest. This is also the most delicate phase of the whole process, as the solidification of the plaster can limit the respiratory exits of the patient, with a rare risk of syncopal events (the frequency of which is directly proportional to the age of the patients). At this point, without waiting for a complete solidification of the plaster, we proceed to its removal, subsequently operating a median longitudinal cut over the entire length and two upper cuts at the level of the shoulders (so that the removal of the same is very easy). Avoiding the complete solidification of plaster brings two major advantages:

• The frequency of syncopes in patients is reduced, as a certain degree of thoracic expansion is still guaranteed.
• The removal of gips can be carried out using simple scissors or blurries with a blurred tip, thus avoiding the use of circular seals for gips.

However, it is precisely the incomplete formation of the plaster that makes this phase of removal very delicate. Therefore, it is necessary to proceed with caution to avoid damaging the rock itself.

At this point, we have completed the stage of realization of the negative model, which is then formed by the gips-calm inside the front part of the shaved shirts with the carenature of the patient.

This model will now serve as the basis for reproducing, always in gips, the bivalve brace of the patient. First, we repair any discontinuities of the negative model by applying additional plaster at the level of the incisions that we had made for its removal. Then we still use plaster to close the opening at the levels of the head and limbs. In this way, we will see that the negative pattern has a single opening, i.e., the lower one.

After modifying the negative pattern, its internal walls are sprinkled with talc, which will serve us in the next stage to separate the negative and positive patterns. Also pertinent to the subsequent stages of the process will be the preparation of an iron rod that is sprinkled with fat and inserted into the negative model through its lower opening. At this point, we can proceed with the implementation of the positive model. The limestone of the patient is placed in a 180°-rotated plaster so that it exposes its lower opening. In the meantime, the plaster powder is immersed in water and mixed to create a homogeneous solution of liquid plaster that will be glued inside the negative pattern. We insert the iron stick inside the negative model, ensuring to keep it as much as possible at the center of the bivalve brace itself as we proceed with the casting of gips. After completing this process and filling the entire bivalve brace with liquid plaster, we wait 15–20 min for the compound to solidify, and we cover with another plaster any discontinuities of the negative pattern. Having reached complete solidification, the plaster bivalve brace is removed from the bathtub, the iron stick is pushed to the opposite end by drilling the upper opening (and the fat served precisely to facilitate this operation), and it is housed and fixed on a special rotating support that allows us to operate the remodel of plaster while keeping it suspended at the height of man.

First, a plate is inserted on the bottom of the bivalve brace, at the level of the hole left free from the stick, to prevent the orthopedic technician from lubricating with the fat during processing operations. At this point, the negative model is separated from the positive, since for the realization of the sternal pressure, we must work exclusively on the latter, which corresponds to the exact reproduction of the chest of the patient. On the positive model should be imprinted the patient's charring previously drawn on the mesh (although sometimes, if not sufficiently stitched, it may not appear because it is “deleted” by the talc itself. In these cases, it is good to always keep in mind the negative model that still keeps the net drawn inside it. After removing the negative model, we can finally work on the positive by proceeding with its remodeling. It is at this stage that the previous objective assessment of the correction margin is important. The remodeling is based primarily on the removal of material from the front and, in the least part, the rear faces to have a slightly narrower layer than the chest of the patient (the greater the correction margin, the more plaster we can remove from the positive pattern). In these operations, it is necessary to follow the principle that “both material is removed before and after, and so much material must be added on the sides of the positive model.” If I removed it exclusively without adding anything, the final result would be a sternal pressure too small for that patient, while following this principle, in the end, I will have a brace of a suitable size that can compress the carotid. The final result of the remodeling will therefore be a positive pattern that, compared to the chest of the patient, will prove to have a smaller anteroposterior diameter and a larger transverse diameter. The last step of this phase is to lighten the entire surface.

After completing these steps, we can finally move on to the realization of the bivalve brace, starting with the processing of the Vivak plates. The first step involves outlining both the valves of the brace with a pencil directly on the positive model, which will guide us in obtaining a brace of the desired size.

In the meantime, we prepare two transparent sheets of plastic on which we will first repaint the drawings of the two valves and then cut those repaints so that we have the exact size of each valve and understand how much Vivak we need to cut. Now, putting the cut plastic sheets on the Vivak plate, we will draw on it the contours of such sheets. Now, with an electric seal, we will cut Vivak so that we can get the two valves of our orthopedic brace. In doing so, we will obtain two plates of a slightly larger size than the end valves, leaving us a small margin of error that can be resolved later in the final assembly phase.

At this point, we proceed to the modeling of the valves, an operation that requires heating in an oven of the same until it reaches 135°C. At these temperatures, the Vivak becomes highly malleable, thus allowing us to obtain the desired morphology, so that the valve, from rigid plates, assumes a conformation that reflects the chest of the patient.

As we wait for the oven to reach temperature, we cover the positive model with a mesh and position it on a second support. This support is equipped with a series of belts anchored at its base, which will be used to shape the valves. In addition, we coat the brace itself with a net mesh; as soon as the valves are baked, their temporary malleability must be exploited. They are then placed on the positive pattern, ensuring they adhere as close as possible to it (the mesh serves to avoid direct contact between the gips and the valve, which is so hot that it would damage the limestone itself). Both the baking and subsequent remodeling of the valves must be carried out separately for each valve; therefore, we proceed first with the front valve (which, once baked, must be molded to the front of the positive model) and then with the rear valve. We try to position the valves as accurately as possible according to the pencil drawing that we have previously made on the gips. As soon as we have given the correct conformation to the valve, we wrap it with the belts placed at the base of the support and stretch them as much as possible to the valve itself to increase its adherence to the positive model. This procedure should be carried out in a short time, as the Vivak takes a few minutes to cool and solidify, leaving us a short window of time to conform to the positive model. At complete solidification, the straps are sliced, and we take advantage of the transparency of the Vivak to recalculate the design of the pressure we had made with a pencil on the plaster. This allows us to understand how much excess Vivak we have used and therefore how much we need to remove to have a valve of the exact size. After completing the remodel and cutting off the excess Vivak, we finally got the valves for our sternal pressure, which only have to be passed to the tape and abrasive paper to smooth the sharp edges. At this point, our front and rear valves are ready, and you only need to assemble them in a single brace: the holes (two on each side) are operated on both valves, the side belts are inserted, and the velcro fittings are attached. At this point, our sternal pressure is complete and assembled, and we must bring the patient back to visit to put a pad on the inner side of the front valve. This device will take the form of the chest of the patient and will serve to amplify at that point the push printed by the pressure itself. If we used the 3D scanner to obtain the chest shape of the patient, the procedures of remodeling and building the valves would be exactly similar, but we should first clean the image scanned to the computer and then give the digital information to the robotic mill, which will work (to subtract material) a cylindrical block of polyurethane. From this, we will get the positive model that, exactly as seen for the classic mode, will be first remodeled and then used as a mold to make the two valves of our sternal pressor. However, as already mentioned earlier, since the remodeling of polyurethane is more complex than gips, the overall time for the realization of the brace is extended, and, to date, the classic gips mode is still preferable.

## Results

The data from the sample examined were analyzed, revealing an epidemiological level with a clear predominance of males (87.1%) over females (12.9%). This conformation of the study sample is consistent with the opinions generally in the literature. Given the peculiar natural history of pectus carinatum, characterized by a gradual and progressive deterioration of the deformity during puberty, and given the need to intervene when there is still good breast elasticity, great importance has been given to the age of the patients at the first visit for a successful conservative treatment. This value ranged between 6 and 22 years, with an average age at the first visit of 13.5 years. This result had a positive impact on the outcome of the patients, as most of them started conservative therapy when chest malleability was still preserved and before the defect worsened to such an extent that it could not be corrected by an orthopedic tutor. Similarly, we analyzed the same data, stratified by gender to see if there were significant differences between the two sexes about the age of presenting the sternal defect. In light of the data, we can say that pectus carinatum, manifesting more or less early during childhood, progresses during puberty and becomes, regardless of gender, aesthetically relevant, especially at the age of 12–14 years (the age group in which the majority of the male and female population examined at the first control visit). Therefore, in light of the data analyzed, it can be stated that there is no statistically significant gender discrepancy regarding the age of manifestation of the malformation. Referring to the various phenotypical manifestations of pectus carinatum, we have obtained data inconsistent with the epidemiology in the literature. Although, according to it, we found a net predominance of the chondrogladiolary variant (94.3%) compared to the condromanubrial (2.1%) and the mixed forms excavated-carenated (3.6%), we did not find any results regarding the division according to symmetry. In particular, all the studies analyzed a higher incidence of symmetrical forms, while in our study sample, we witnessed 58.6% of asymmetric forms (prevailing right) and 41.4% of symmetric ones. Further evidence showed a clear predominance (99.3%) of isolated forms over syndromic forms (0.7%). Among the population studied, only one patient suffered from a syndrome that can sometimes be associated with pectus carinatum. Specifically, this was a male patient with Marfan syndrome, who benefited from a new brace that put more pressure on his defect after an initial therapeutic inefficiency, achieving a significant improvement in the chest profile (also linked to excellent compliance with therapy).

In general, the outcomes considered in the evaluation of the study results are:

• compliance with therapy;
• aesthetic result after a minimum of 6 months of follow-up; and
• the possible occurrence of complications associated with the use of the custom-made bivalve brace.

The data analysis revealed a remarkably high level of adherence to therapy, which is particularly significant considering that, since the 1970s, the main etiological factor of therapeutic failure has always been the lack of collaboration among patients. Despite the proposal of a rigid brace instead of a dynamic compression system, the properties of Vivak have still made compliance easier for patients. In particular:

• the lightness of the bivalve brace favors the maintenance of the same throughout the days, without excessive burden on patients; and
• the reduced thickness (3–5 mm) of the valves themselves favors their covering under clothing.

In more detail, there were no cases of absolute non-compliance with the therapeutic plan, and cases of poor compliance represent a clear minority (5.1%) of the analyzed sample, while 94.9% of patients stated that they faithfully follow the prescribed treatment protocol, with obvious consequences on the outcome of treatment.

Overall, these compliance results are also confirmed by the quality of life questionnaire filled out by the patients themselves, whose average value on a scale from 0 to 10 for device tolerability was 8.6. Further confirmation is given by the fact that there have been no cases of abandonment of the experimental study, further supporting the hypothesis that the brace is better tolerated than the devices present in the literature.

In relation to the outcomes of therapy, clinical improvement was observed in 100% of the sample population, reaching complete resolution of the malformation in 34.8% of cases. Furthermore, these results were achieved regardless of the type of pectus of the subjects, demonstrating the absolute effectiveness of the subject brace in all forms of pectus carinatum, unlike all dynamic compression devices in the literature that were ineffective in most asymmetric chondrogladiular, condromanubrial, and mixed deformities. In particular, the effectiveness of the custom bivalve brace was analyzed by distinguishing the sample into symmetrical and asymmetric forms, resulting in that:

• therapeutic success, in terms of improvement of the clinical picture, was observed in 100% of patients, regardless of the symmetrical morphology or not of the defect;
• in symmetrical forms, the complete resolution of the defect occurred in 41.1% of cases.

In asymmetric cases, the resolution involved 30.5% of patients.

Therefore, a greater effectiveness of conservative therapy in symmetrical forms than in asymmetric forms persists, leading to resolution in a higher percentage of cases. Nevertheless, in any case, the bivalve brace did not lead to a noticeable improvement in the chest profile.

**Figure 4:**
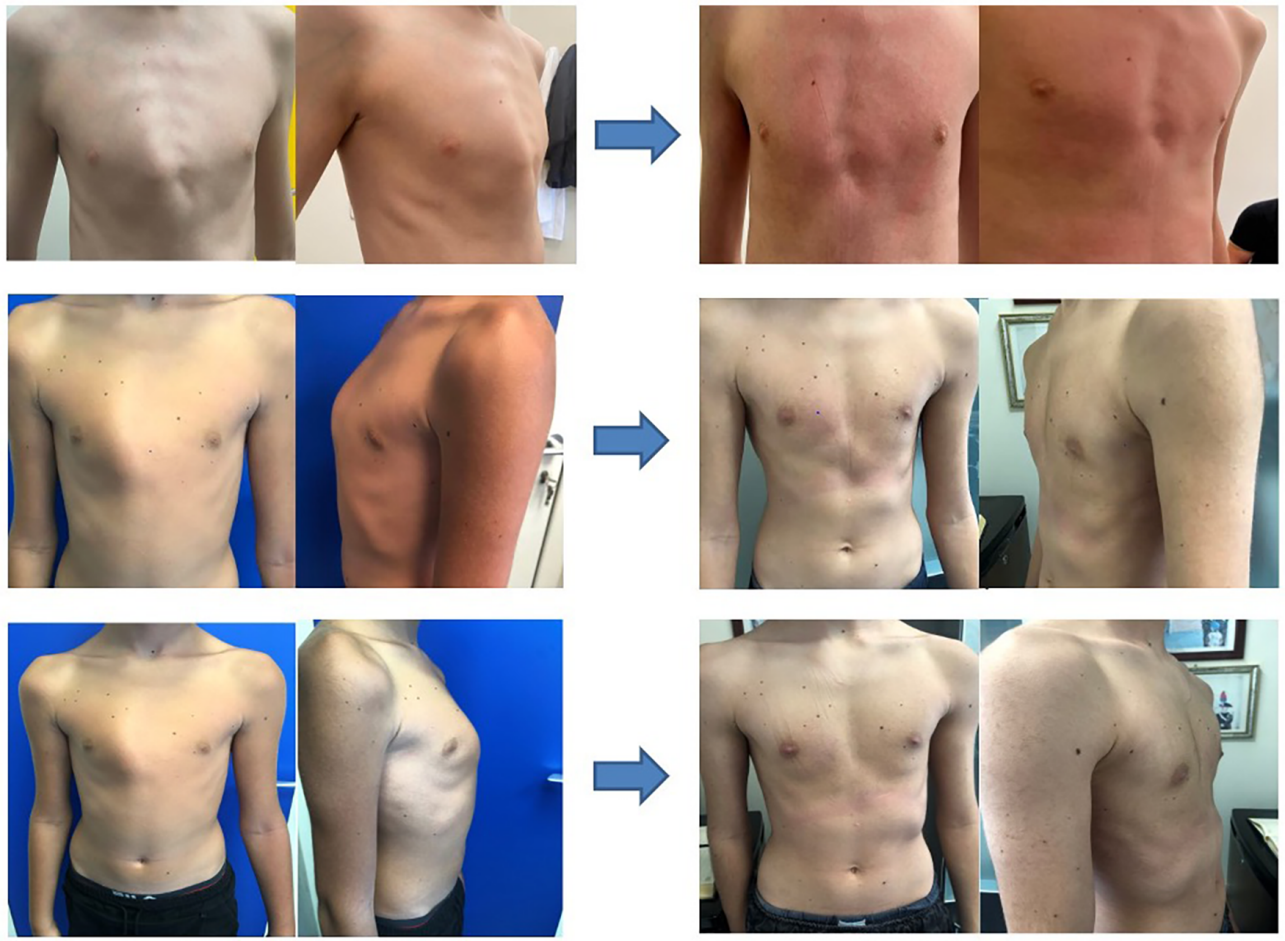
Examples of pre- and post-treatment.

The results of these analyses show the exceptional effectiveness of the experimental brace, confirmed both by the questionnaire carried out by the patients (with an average satisfaction rate of 8.9/10) and by a properly selected medical committee to assess the results of the therapy itself (through a VAS scale, their satisfaction was 7.2/10 for symmetric forms and 7.1 for asymmetric ones) (Figure 4).

In reference to the last of the three outcomes examined, i.e., the possible occurrence of complications associated with bivalve brace therapy, there were no cases of erythema, skin lesions, costal flaring, excessive sweating, or dyspnea related to the use of the device. The only evidence, in a minority of patients, of a region of redness in the area where the brace prints the push in the front–back direction is the only clinical relief to be noted, which is not considered as a complication but rather as a sign of confirmation of the compliance of the patients and the effective corrective action to bring back the sternum and ribs to their natural position. During the 2 years of treatment, the patient is checked every 6 months. The brace is modified on average two or three times, based on the growth of the chest of the patient.

## Discussion

In this article, we discussed our early promising outcomes with 140 patients using a novel personalized (3D scanner) bivalve brace for non-operative management of pectus carinatum in children and adolescents. This device is made of Vivak, a plastic material that combines exceptional thermoplastic properties. The material, which is used to create the front and back valves of the brace, is light and thin while still having shock and UV ray resistance. Before 2020, we used the same type of brace, and the difference is in the length. The previous model covered the entire trunk up to the iliac spine, resulting from our experience in the treatment of scoliosis. The model currently in use, covering exclusively the thoracic region affected by the malformation, is certainly better tolerated by patients and more easily concealed under clothing.

The best way to manage pectus carinatum is still up for debate due to the rarity of the anomaly and the paucity of information. There are currently no guidelines or consensus on the subject. Numerous techniques have been reported since the first account of surgical repair by Dr. Ravitch in 1952 (3). The currently known operative techniques may employ different strategies and/or approaches. Some of the surgical procedures needed a varying amount of costal cartilage removed. Various approaches can be used to perform the resection: open (3–6) or thoracoscopy (7–9). Otherwise, several non-resectional procedures have been described and reported (10–14).

Instead, the first isolated reports related to non-operative management were described in the 1960s and involved bracing systems. However, the revolutionary concepts independently proposed by Dr. Jaubert de Beaujeu in 1964 (published in French) and Dr. Bianchi in 1968 (published in Italian) went unnoticed and unapplied for decades (15, 16). Since the 1990s, the concept of this progressive thorax reshaping has been rediscovered and diffused. Indeed, in 1992, Dr. Haje et al. reported the first successful series of patients with pectus carinatum treated non-operatively (17). These authors elaborated on a new device called dynamic chest compressor (DCC). Since this first report, the approach diffused progressively worldwide in the following decades and today non-operative management is considered the first-line option for this anomaly (18). Many benefits of the conservative approach contributed to this shift in practice. First off, there is no need for hospitalization, which lessens the burden on patients and families as well as cuts costs. Furthermore, if a non-operative approach is unsuccessful, a first non-operative treatment does not rule out a subsequent second-line surgery. Furthermore, non-operative treatment avoids the risks and complications of surgery and general anesthesia. The main problem with bracing was the tolerance of the patients.

Another important aspect to consider is the cost-effective accessibility of the bivalve brace, a requirement not always met in the various dynamic compression models created over the years and mentioned in the study. With regard to these assumptions, much of the experimental study focused on evaluating two aspects, patient compliance and therapeutic outcome, and the close relationship between them. Overall, 94.9% of patients wearing the brace regularly as suggested at the first visit (i.e., keeping it in place for at least 23 h a day for the duration of therapy), without any case of discontinuation of the study. This data on patient compliance is at least surprising if you consider that throughout the literature examined (15–19), there has always been a high rate of failure due to the unsustainability of therapy and therefore a high percentage of reduced compliance or even abandonment of the study.

As a result, the aesthetic results were also evident (and in 34.8% of cases also excellent) in all patients, regardless of the severity and type of pectus carinatum they were affected by.

In conclusion, based on the data analyzed, it can be stated that the research hypotheses have been confirmed. First, it was found that in none of the 140 patients, there were cardiovascular diseases directly related to their condition, thereby confirming that pectus carinatum is a pathology of a purely aesthetic nature. Second, we were able to verify that a cheap, lightweight, and easily obscured brace significantly improved the compliance of patients, and this, along with the use of a rigid brace that guaranteed greater effectiveness than a dynamic compressor, led to a high number of positive outcomes.

Together with this, an aspect not to be neglected is the social relevance that the aesthetic aspect today holds, which could be an important factor in support of the motivation demonstrated by the study cohort in adhering to therapy. It is also likely that in the past, esthetics and appearance were less relevant elements at the social level, ending up affecting greatly the high rates of abandonment and reduced compliance of the numerous studies in the literature.

## Data Availability

The original contributions presented in the study are included in the article/Supplementary Material, and further inquiries can be directed to the corresponding author.
